# A comparison of camera trap and permanent recording video camera efficiency in wildlife underpasses

**DOI:** 10.1002/ece3.3149

**Published:** 2017-08-11

**Authors:** Jonathan Jumeau, Lana Petrod, Yves Handrich

**Affiliations:** ^1^ Université de Strasbourg CNRS IPHC UMR 7178 Strasbourg France; ^2^ Conseil Départemental du Bas‐Rhin Place du Quartier Blanc Strasbourg Cedex 9 France; ^3^ Université de Rennes 1 CNRS UMR 6553 ECOBIO Rennes Cedex France

**Keywords:** camera trapping, false trigger, monitoring study, small mammals, triggered cameras, wildlife crossings

## Abstract

In the current context of biodiversity loss through habitat fragmentation, the effectiveness of wildlife crossings, installed at great expense as compensatory measures, is of vital importance for ecological and socio‐economic actors. The evaluation of these structures is directly impacted by the efficiency of monitoring tools (camera traps…), which are used to assess the effectiveness of these crossings by observing the animals that use them. The aim of this study was to quantify the efficiency of camera traps in a wildlife crossing evaluation. Six permanent recording video systems sharing the same field of view as six Reconyx HC600 camera traps installed in three wildlife underpasses were used to assess the exact proportion of missed events (*event* being the presence of an animal within the field of view), and the error rate concerning underpass crossing behavior (defined as either *Entry* or *Refusal*). A sequence of photographs was triggered by either animals (*true trigger*) or artefacts (*false trigger*). We quantified the number of false triggers that had actually been caused by animals that were not visible on the images (“false” false triggers). Camera traps failed to record 43.6% of small mammal events (voles, mice, shrews, etc.) and 17% of medium‐sized mammal events. The type of crossing behavior (*Entry* or *Refusal*) was incorrectly assessed in 40.1% of events, with a higher error rate for entries than for refusals. Among the 3.8% of false triggers, 85% of them were “false” false triggers. This study indicates a global underestimation of the effectiveness of wildlife crossings for small mammals. Means to improve the efficiency are discussed.

## 1 INTRODUCTION

Habitat fragmentation is one of the three main causes of biodiversity loss over the last decades (Maxwell, Fuller, Brooks, & Watson, 2016). It affects all taxonomic groups and has numerous consequences. Habitat fragmentation through habitat loss usually results in a stream of extinctions, the extent of which depends on modified soil surfaces and their organization (Fischer & Lindenmayer, 2007). Fragmentation has a negative impact on the richness, population abundance, growth rate and distribution, trophic chain length, breeding, and dispersal success of animal species. Decreased genetic flows result in increased consanguinity and genetic drift, leading to the risk of local species extinctions (Fahrig, 2003). Habitat fragmentation *per se* has a negative impact on the time animal populations spend in the landscape matrix, and affects their interspecific interactions, mortality, and reproduction rates, thus influencing their long‐term persistence. It also has positive effects by stabilizing the prey–predator relationship, permitting the persistence of two species that share a similar ecological niche (competitive species), distributing the extinction risk across habitats and increasing landscape heterogeneity. However, the positive effects of fragmentation *per se* are less significant than the negative effects linked to habitat loss (Fahrig, 2017).

The main cause of habitat fragmentation is urban expansion. Roads have a specific impact and cause wide‐ranging damage to ecosystems, ranking in the top 10 threats to biodiversity (Maxwell et al., 2016). In the short and/or long term, roads increase local extinction risk through wildlife–vehicle collisions, lead to further habitat loss due to associated land consolidation, deteriorate habitats along the roadside, decrease population abundance (avoidance phenomenon), and reduce genetic flows by creating a filter and barrier effect. According to Ibisch et al., 2016, 20% of the world's surface is located within a 1‐km road‐effect zone, including the vast majority of the land in Western Europe and the Eastern United States. Roads do, however, have positive effects, such as increasing longitudinal dispersion flows and creating road verges that are used as substitute habitats by a variety of taxa (Benítez‐López, Alkemade, & Verweij, 2010; Forman & Alexander, 1998; Ibisch et al., 2016; van der Ree, Smith, & Grilo, 2015; Rytwinski & Fahrig, 2013; Spellerberg, 1998; Trombulak & Frissell, 2000).

The negative impacts of roads are reduced by mitigation measures such as fences and wildlife crossings. The latter are bridges or tunnels that provide a safe crossing point for wildlife, thus restoring genetic exchange and ecological processes (Clevenger, 2005). Wildlife crossings have been constructed over the last five decades and are adapted to targeted species. Among the different types of wildlife crossings (Beben, 2016; Forman et al., 2002), underpasses for small‐/medium‐sized wildlife have a small entrance and are adapted to small animals such as rodents and mesocarnivores (Carsignol et al., 2005; Clevenger & Huijser, 2011; Martinig & Bélanger‐Smith, 2016). Each wildlife crossing is carefully designed to encompass technical constraints, local environment, and species needs. Factors influencing the effectiveness of wildlife crossings include openness ratio (Navàs & Roselll, 2005), location (Land & Lotz, 1996), local roadkill mitigation measures (Dodd, Gagnon, Boe, & Schweinsburg, 2007), funnel devices (Ascensão & Mira, 2007), entrance design (Rodrigez, Crema, & Delibes, 1996), type of vegetation cover (McDonald, Cassady, & Clair, 2004), human activity (Clevenger & Waltho, 2000), and traffic (Clevenger & Waltho, 2000). By monitoring the use of wildlife crossings by animals, environmental actors can identify the needs of animals and improve these structures (van der Ree, Jaeger, Rytwinski, & van der Grift, 2015).

The first monitoring studies of wildlife crossings in Europe, Australia, and North America used sand boxes to observe animal tracks, and particularly those of big game (Forman et al., 2002). More sophisticated methods are now employed, such as infrared or thermal video cameras (Serronha, Mateus, Eaton, Santos‐Reis, & Grilo, 2013), genetic sampling (Corlatti, Hackländer & Frey‐Roos, 2009; Sawaya, Kalinowski, & Clevenger, 2014), GPS collars (Dodd, Gagnon, Boe, & Schweinsburg, 2005; Olsson & Widen, 2008), radio telemetry (Baxter‐Gilbert, Riley, Lesbarrères, & Litzgus, 2015; Dillon & Kelly, 2008), acoustic, infrared and microwave sensors (Diggins, Gilley, Kelly, & Ford, 2016; Glen, Cockburn, Nichols, Ekanayake, & Warburton, 2013; Gužvica et al., 2014), and automatically triggered cameras, also called camera traps (Šver, Bielen, Križan, & Gužvica, 2016). The choice of a monitoring technique depends on the targeted species, the goal of the study and the human and financial investment (Hardy, Clevenger, Huijser, & Neale, 2003). The effectiveness of wildlife crossings commonly depends on the number of species detected by the monitoring methods, and is therefore highly dependent on the efficiency of the latter. It is therefore necessary to have a critical approach and to know the drawbacks of each method, particularly for the most commonly used method, the camera trap. This method is popular for its easy installation and use and its relatively moderate cost. It is also a relatively non‐intrusive method (Meek & Vernes, 2016).

The efficiency of a monitoring method can be defined as its ability to detect the greatest proportion of species in relation to its global cost, including the time invested in the installation, maintenance, and data analysis process (Mateus, Grilo, & Santos‐Reis, 2011). In order to estimate the efficiency of camera traps, some studies compared this method to others (Diggins et al., 2016; Dillon & Kelly, 2008; Glen et al., 2013; Janečka et al., 2011; Li, McShea, Wang, Huang, & Shao, 2012; Lyra‐Jorge, Ciocheti, Pivello, & Meirelles, 2008; Monterroso, Rich, Serronha, Ferreras, & Alves, 2013; Silveira, Jácomo, & Diniz‐Filho, 2003; Villette, Krebs, Jung, & Boonstra, 2016). Other authors compared different models of camera traps (Hughson, Darby, & Dungan, 2010; Meek & Vernes, 2016; Rovero, Zimmermann, Berzi, & Meek, 2013; Swann, Hass, Dalton, & Wolf, 2004; Weingarth, Zimmermann, Knauer, & Heurich, 2013), their technical parameters (Kelly & Holub, 2008; Pease, Nielsen, & Holzmueller, 2016), and the different installation and placement methods (Foster & Harmsen, 2012; Guil et al., 2010; Smith & Coulson, 2012). As the efficiency of camera traps also depends on the targeted species and their characteristics (Ariefiandy, Purwandana, Seno, Ciofi, & Jessop, 2013; Lyra‐Jorge et al., 2008; Rowcliffe, Carbone, Jansen, Kays, & Kranstauber, 2011; Tobler, Carrillo‐Percastegui, Leite Pitman, Mares, & Powell, 2008; Welbourne, MacGregor, Paull, & Lindenmayer, 2015), the use of lures (Diete, Meek, Dickman, & Leung, 2016; MCCleery et al., 2014; Read, Bengsen, Meek, & Moseby, 2015) and the associated bias (Meek et al., 2014b; Newey et al., 2015; Rocha, Ramalho, & Magnusson, 2016) were also investigated. However, to our knowledge, no study to date has compared methods and parameters by taking into account the exact number of animals present in the camera trap field of view. The systematic recording of all animals using an underpass can be achieved through permanent video recording of the camera trap field of view. The time required to analyze data is another parameter of camera trap efficiency, and relevant software packages have been developed to improve this (Bubnicki, Churski, & Kuijper, 2016; Fegraus et al., 2011; Niedballa, Sollmann, Courtiol, & Wilting, 2016; Yu et al., 2013). Time is not only allocated to the examination of photos and videos, but is also needed to identify and classify false triggers (Glen et al., 2013). Triggers can have different sources. They are considered “true” when animals trigger photo sequences, and “false” in all other cases. However, some of the latter could be still due to an animal that is not visible in the photo. This is a type of false‐negative, and could be termed a “false” false trigger (Meek et al., 2014a). The proportion of these missed events has not been evaluated to date.

After a long period dedicated to the monitoring of big game, monitoring interest has now turned to small fauna and small mammals, which now represent a significant portion of the species studied with camera traps (Burton et al., 2015). Their detection is difficult due to their low body weight (Di Cerbo & Biancardi, 2012; Diete et al., 2016; Diggins et al., 2016; Glen et al., 2013; Ikeda et al., 2016; MCCleery et al., 2014; Meek, Ballard, Fleming, & Falzon, 2016; Meek & Vernes, 2016; Melidonis & Peter, 2015; Rendall, Sutherland, Cooke, & White, 2014). The effectiveness of wildlife crossings for these species, previously estimated by tracking beds and live trapping, is increasingly studied with technologically advanced camera traps (Bellis, Jackson, Griffin, Warren, & Thompson, 2013; Martinig & Bélanger‐Smith, 2016).

The aim of the present study was to quantify the efficiency of small fauna detection by camera traps installed in underpasses for small‐/medium‐sized wildlife, using a permanent recording camera system as a control recording. The specific questions of this study were as follows: (1) How many events are not detected by camera traps, and which parameters are involved? *Events* are defined here as the presence of an animal within the camera trap field of view. (2) What is the error rate of camera traps in the estimation of underpass crossing behavior (*entry* or *refusal*), and which parameters are involved? (3) Among the false triggers, what is the proportion of “false” false triggers and “true” false triggers?

## 2 METHODS

### 2.1 Study area

The study was performed in the Bas‐Rhin (Eastern France, GPS coordinates: 48.517256, 7.582047) on the A35/A352 trumpet interchange, which is 2‐km long and was opened to traffic in 2010. The studied section was a two‐lane fenced highway with average traffic of 22,000 vehicles per day. The interchange was surrounded by intensive agricultural landscape dominated by maize and wheat fields. The 2‐m high fence was composed of wire netting to exclude small mammals on the lower 50 cm (6.5 mm mesh) and a large mammal exclusion fence on the upper section (12‐cm mesh). The interchange was equipped with 11 underpasses (three agricultural and eight underpasses for small‐/medium‐sized wildlife) at its construction, installed approximately 230 m apart. The mean annual temperature of the studied area is 10.4°C. Mean annual precipitation is 700 mm per year, at a mean altitude of 150 m.

### 2.2 Protocol

#### 2.2.1 Studied underpasses

Three small‐/medium‐sized wildlife underpasses (hereafter referred to as “WU”s) designed for the European hamster (*Cricetus cricetus*) were monitored. These concrete box culverts contained a 10‐cm soil bed. Their characteristics, including the openness ratio (OR = Width*Height/Length; Reed, Woodard, & Pojar, 1975 in Donaldson, 2005) are described in Table 1.

**Table 1:** Wildlife underpass characteristics

Underpass *N* °	OR (m)	Length (m)	Width (m)	Height (m)
1	0.01	51	1.2	0.5
2	0.01	50.4	1.15	0.75
3	0.02	24	1.2	0.5

#### 2.2.2 Monitoring material

To perform the monitoring, camera traps and permanent recording video systems were used simultaneously in each WU. The camera traps were all Reconyx HC600 HyperFire models bought new in 2012. This middle‐range priced model has a passive infrared sensor and uses infrared LEDs to reduce glow. Sensitivity was set to “high,” and “still mode” was used. A sequence of five photos was taken at 0.5 s intervals, with no delay between sequences. Trigger speed was 0.2 s and image resolution was 3.1 megapixels. Camera traps were supplied with non‐rechargeable lithium batteries. The permanent recording video system was composed of CGV DFAV3312JN video cameras, a STIM ST4100 video server for recording and additional Bosch EX12LED 940 nm infrared lights. The 940 nm wavelength was chosen for its low detectability by small wildlife in the studied area (Meek et al., 2014b; Newbold & King, 2009; Odonat: Office des données naturalistes du Grand‐Est 2016). The video system was set up for permanent recording at 40 Hz with a 752 × 582 image resolution.

#### 2.2.3 Installation of material

We installed the permanent recording video systems from 03/26/2012 to 04/27/2012, and set up the camera traps on 04/27/2017. The two different device types were installed in both entrances of the three WUs to ensure that all evidence of animal presence would be captured by the permanent recording video systems when entering the WUs, even if individuals were not detected by the camera traps. A total of six tested camera traps and permanent recording video systems were therefore installed for the monitoring of three WUs.

Camera traps were installed 2 m from the entrance of each WU, and were attached to a sliding rail on the ceiling to permit the maintenance of the camera outside the WU. This method was used to limit the presence of human scent on both the monitoring devices and the entrance of the WU. The fixed distance allowed camera traps and permanent recording video systems to cover the entire WU field. A 2‐m distance is well adapted to the observation of small wildlife (Rowcliffe et al., 2011).

Camera traps were turned toward the underpass entrance, and were suspended 19.5–44.5 cm above floor level (distance between bed soil and passive infrared sensor) and oriented about 10 degrees downwards to view the first meter of the underpass. Camera traps were perpendicular to the ground, so the sensor was placed horizontally (Smith & Coulson, 2012; Taylor, Goldingay, & Lindsay, 2014). Devices were placed on an east‐west axis, directly exposing them to sunlight in the mornings and evenings. The vegetation at WU entrances was not mowed during the study.

**Figure 1:**
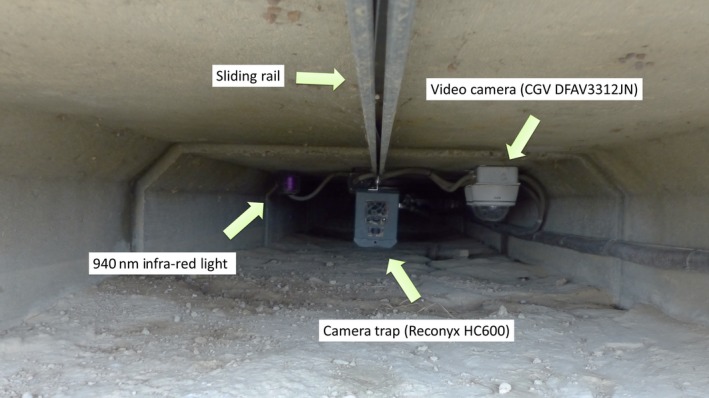
Relative positions of camera trap, video camera, and infrared light

**Figure 2:**
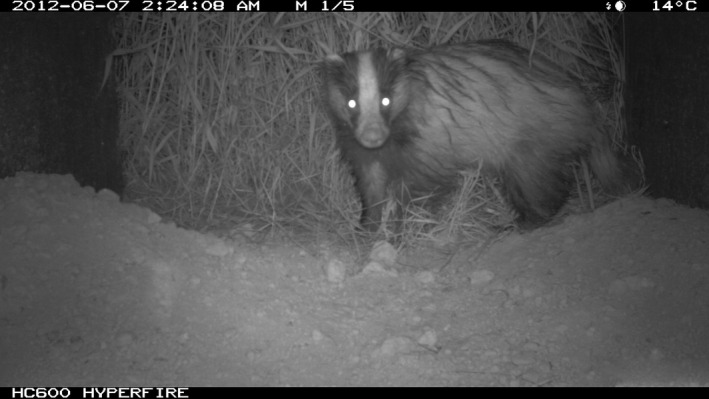
Camera trap and video camera field of view

Permanent recording video systems were fixed to the ceiling 2 m from the entrance and 18–43 cm from the ground (distance between the ground and camera lenses). Infrared lights were installed on the ceiling at the same distance from the entrance, 21–44 cm from the ground (distance between the ground and the middle of LEDs). A video camera and an infrared light were set up 20 cm to the left and right of each camera trap, respectively (video on the right and light on the left). The infrared lights were installed at a distance from video cameras to prevent the occurrence of spider webs on the camera lens. Video cameras were oriented in the same direction as that of the camera traps in order to obtain an identical field of view. The inclination and orientation of the infrared lights were adjusted between 12 a.m. and 4 a.m. on 04/26/2012 to maximize the lighting of the area covered by both cameras through the use of light reflection on underpass walls. The positioning of camera traps and permanent recording video systems is shown in Figure 1. The camera trap field of view is shown in Figure 2. Each video camera and light for the permanent recording video system was connected to central units that were located outside the underpass and equidistant between two wildlife crossings, providing power and memory via electric cables and optical fibers. Cables were buried outside underpasses and attached to the wall 15–40 cm from the ground inside WUs.

#### 2.2.4 Data collection

Permanent recording video systems and camera traps initially recorded from 22/05/2012 to 09/07/2012. A second record session was performed from 30/05/2013 to 17/07/2013 (64 days in all) to increase the sample size. Camera trap batteries and memory cards were checked and replaced by the same person every 2 weeks throughout the study. At the same time, the video server containing video recordings was replaced and the data collected from the previous server was analyzed at the laboratory.

### 2.3 Analysis

#### 2.3.1 Camera traps

In the present study, an *event* is defined as the presence of an animal (a given individual of a given species) in the field of view of both camera traps and video cameras in a given entrance of a given WU (Meek et al., 2014a). All events were recorded by the permanent recording video system but not necessarily by camera traps. To decrease individual bias, the event delineator was set at 10 min for all species (Meek et al., 2016; Silveira et al., 2003). Thus, if any two animals of the same species were recorded <10 min apart in the same entrance of the same WU by the permanent video recording system, the same individual was considered to have been seen twice. Only the first of the two events was retained in the dataset. If two individuals were clearly different in terms of body size, age, state of health, and morphologic characteristics, the event delineator was not applied (Read et al., 2015). When two species or several individuals of the same species were present in the photo, different events were created for each individual, with no event delineator.

For each camera trap and event, two crossing behaviors were distinguished: “Entry” occurred when the individual was seen entering the underpass and left the camera field of view, which means that the animal walked at least 1 m inside the underpass without be seen again; if the individual did not do so, “Refusal” occurred. Short events (≤3 s) involving fast animals were separated from longer events (>3 s).

Photos were analyzed using the DREAL OCR software (v1.5.5) developed by Pierre Charbonnier and Stéphane Sadowski from CEREMA‐LRS. This semi‐automatic analysis software is specifically designed for use with Reconyx HC600 Cover and Hyperfire. It extracts all the EXIF information from the photos, can correct trigger time according to a reference in order to synchronize all the camera traps, uses an OCR system to extract all the information that is not in EXIF format, and greatly facilitates analysis with easy use and “one‐click” species recognition.

Data for the following were collected from sequences of photos: date, underpass number, underpass entrance, camera trap number, time to the nearest second, temperature, species, crossing behavior (*Entry* or *Refusal)*, event duration, and the direction of the animals (*Horizontal* or *Vertical*) observed on each sequence of photos. The occurrence of false triggers was also noted. All photos were analyzed by the same experimenter.

#### 2.3.2 *Video camera*

Videos were analyzed at ×5 speed, that is, slow enough to allow the experimenter to detect any change in a sequence of similar photos and fast enough to optimize analysis time. The speed was changed back to ×1 if an event was detected or if a false trigger of camera traps occurred. We used the recording server analysis software. Data were collected for the same variables for both camera traps and videos, but videos analyzes were performed by two different experimenters after completing a similarity test based on the observation at ×5 speed of 3 hrs of video containing 10 events whose data (time, species, crossing behavior, event duration, and direction of the animals) had to be analyzed exactly the same way between the two experimenters.

#### 2.3.3 Data analysis and statistics

To study the efficiency of camera traps, we compared different event characteristics (number, crossing behavior, and false triggers) with those of the corresponding events recorded by the permanent recording video system. “Small mammals” were distinguished from “Medium‐sized mammals.”

To avoid a violation of data dependence due to the same animal crossing the same underpass, that is, recorded by both camera traps, only one of the two observations (entry or exit) was randomly taken into consideration for analysis.

We used the following dependent variables: (1) Did the camera trap detect the animal? (*Yes/No)*; (2) Was the crossing behavior the same for camera trap and permanent recording video system? (*Yes/No*; assuming that the video depiction of the behavior is reliable); (3) Was the false trigger due to an animal or to a trigger artefact? (“false” false trigger/“true” false trigger).

Binomial Generalized Linear Mixed Model (GLMβ) based ANOVAs were used to explain the variations of these dependent variables. Co‐variables were as follows: animal direction on the photos (*Horizontal* or *Vertical*), event duration (*≤3s or >3s*), animal size (*Small mammals* or *Medium‐sized mammals*), and crossing behavior (*Entry* or *Refusal*). Statistics were performed with R version 3.2.5. The significance threshold was set to 5%.

## 3 RESULTS

### 3.1 Collected data

A total of 8,415 photos and 9,234 hrs of videos were recorded over 64 nights, making a total of 384 trap nights for all the camera traps. There was no failure or device theft. During the test periods, the temperatures in the studied underpasses varied between 9 and 22°C, with a mean of 15.6°C (±2.4°C *SD*).

A total of 13 vertebrate species were detected, of which 85% were mammals. Non‐mammalian species were discarded from our data (white wagtails and house sparrows). The remaining 11 mammal species were separated into two groups according to their size. A “medium‐sized animal” group pooled hares (*Lepus europaeus*) and several carnivores, namely the red fox (*Vulpes vulpes*), the European badger (*Meles meles*), and the domestic cat (*Felis silvestris catus*). The other mammalian species were pooled in a “small‐sized animal” group composed of the hedgehog (*Erinaceus europaeus*), the weasel (*Mustela nivalis*), the wood mouse (probably *Apodemus sylvaticus*), voles (*Microtus agrestis/arvalis*), the European water vole (*Arvicola amphibius*), the brown rat (*Rattus norvegicus*), and shrews (probably *Crocidura sp*.).

### 3.2 Recorded events

A total of 747 triggers containing 683 events were recorded by permanent recording video systems before the application of a 10‐min event delineator. Among the remaining 237 events (225 small mammals and 12 medium‐sized mammals), only 137 (57.8%) were also detected by camera traps after application of the 10‐min delineator.

There was a tendency for a lower detection of small mammals in comparison to that of medium‐sized ones (χ² = 3.636; *df* = 1; *p* = .057); camera traps failed to record 98 small mammal events (43.6%) and two medium‐sized mammal events (17%). Camera traps detected significantly fewer short events (≤3 s), associated with fast animals, than longer events (χ² = 43.813; *df* = 1; *p* < .01). The detection of refusals was significantly more efficient than that of entries (χ² = 7.71; *df* = 1; *p* < .01). The number of undetected events did not depend on the direction of animals (χ² = 1.419; *df* = 1; *p* = .233).

### 3.3 Crossing behavior

Among the 237 events, 91 (38.4%) were entries and 146 (61.6%) were refusals. The number of crossing behavior detection errors (different behavior between camera traps and permanent recording video systems, presuming that the video depiction of the behavior was reliable) was 95 (40.1%). Error risk was significantly higher for entries than refusals (χ² = 6.355; *df* = 1; *p* = .011; 50.54% vs. 33.56%). This risk did not depend on event duration (χ² = 2.63; *df* = 1; *p* = .105), animal size (χ² = 0.03; *d* *f*= 1; *p* = .863), or the direction of animals (χ² = 0.362; *df* = 1; *p* = .547).

### 3.4 False triggers

Among the 747 triggers (8,415 photos), 64 were false triggers (3.8%, corresponding to 320 photos). Among the 64 false triggers, 10 were “true” false triggers (15.625%, corresponding to 50 photos) and 54 were “false” false triggers (84.375%, corresponding to 270 photos), that is to say true triggers by an animal presence that was not recorded on camera trap photos. ANOVAs were not performed for false triggers due to the low sample size.

## 4 DISCUSSION

### 4.1 Factors driving the probability of detection and crossing behavior error risk

Even with the best monitoring protocol using camera traps, and whatever the model chosen, it is impossible to evaluate the percentage of undetected animals crossing a wildlife underpass (WU) without the help of permanent monitoring. The present study used permanent 40 Hz video monitoring to certify all the events that occurred in the field of view of six camera traps, and reveals that 43% of all mammals were missed by camera traps. This result is especially relevant given that the chosen model of camera trap, its parameters and installation as well as the environmental conditions meet the efficiency criteria (see Methods) set out in the available literature (Meek et al., 2016; Rovero et al., 2013; Rowcliffe et al., 2011; Tobler et al., 2008).

The higher detection rate of medium‐sized mammals (in comparison to small mammals) is probably due to their greater body weight (Lyra‐Jorge et al., 2008; Tobler et al., 2008) rather than their lower speed (Rowcliffe et al., 2011). However, short events were harder to detect than longer ones (≤3 s). Some mice were probably accustomed to the WU, ran in a straight line, and thus stayed within the monitored field of view for <1 s. Other small mammals immediately entered and left the wildlife crossing via the same entrance. There was no effect of animal direction (*Horizontal* or *Vertical*) on the detection rate, which shows that the Reconyx double‐bands passive infrared detection system can be efficiently triggered by both animal directions. Refusals were detected better than entries, which could result in an overestimation of refusals and thus an underestimation of underpass effectiveness.

The identification of crossing behavior is necessary to evaluate wildlife crossing effectiveness. A high number of refusals could mean the WU is unsuitable for a given species. Despite the easy distinction used in this study between refusal and entry, a large number of crossing behavior identification errors were made, especially for the entries. Animals entering the WU could leave the camera trap field of view after the detection of their entry, then leave the WU via the same entrance without this refusal being detected by the camera trap. In this situation, the underpass effectiveness would be overestimated.

If the recorded animals are considered representative of the whole population, these results may not call into question the conclusions of previous studies. However, there is a bias due to the high proportion of fast and small mammals among unrecorded events.

### 4.2 How to improve the detection rate and reduce crossing behavior error risk

The detection rate could be improved using lures to make the animals stay longer in front of the camera trap (Diete et al., 2016; MCCleery et al., 2014), but this would increase the probability of a prey running into a predator (Little, Harcourt, & Clevenger, 2002; Tissier et al., 2016). According to Rocha et al. (2016), the use of lures could also decrease the probability of preys animals triggering camera traps.

Another way to improve the detection rate would be the simultaneous use of several camera traps (Pease et al., 2016). This can be easily achieved in the underpasses by placing one camera at each end of them, as we do in this study, rather than one on the middle. Using two camera traps would also allow better accuracy for the recording of crossing behavior. Indeed, if an animal that enters a WU is detected by a second camera trap located at the other end, it would be proof that this animal successfully entered and crossed the underpass. If not, the crossing behavior should be considered as uncertain, thus avoiding an overestimation of underpass effectiveness. The sequential mode (i.e., a photo taken every X s/min) could also be used, as its detection rate is not affected by sensors and species. However, its efficiency remains to be demonstrated for small wildlife, and amphibians in particular (Morand & Carsignol, 2016).

Lastly, the detection rate could be improved by moving the camera trap back a few meters inside the underpass to evaluate if an entry could be a refusal. However, animals that remain outside the underpass would be less detected.

### 4.3 False triggers

Less than 5% of the triggers were false triggers, that is, without any animal presence recorded. Despite being low, this proportion can be problematic when a large number of photos are taken. For example, the monitoring of all underpasses present on the studied area produced 1.5 million photos over 4 years (*data not shown*). Although software that facilitates the removal of the high number of false triggers is available, the latter may contain a large number of “false” false triggers (almost 85% in this study), which could be erroneously removed from the dataset (*false‐negative*; Meek et al., 2014a). To be able to consider them as full events, we therefore have to decrease the number of “true” false triggers. Inside wildlife crossings, camera traps are less prone to disturbances, particularly when vegetation is cleared in front of the underpass to improve visibility or when the camera traps are installed far from the entrance. The high proportion of “false” false triggers we obtained should therefore be considered in a specific context. Abundance monitoring in the natural habitat would show a lower proportion of “false” false triggers, especially if it uses low‐quality devices (e.g., recreational cameras; Newey et al., 2015). To avoid deleting too many “false” false triggers in WU, we should only remove the false triggers recorded when the sun directly hits the passive infrared sensor or when the wind is too strong. The remaining events could be considered as “unidentified animal events.” The high proportion of “false” false triggers also leads to an underestimation of underpass effectiveness.

### 4.4 Is the permanent video camera system a better solution?

The use of a permanent recording video system in this study allowed us to quantify some aspects of camera traps efficiency. Video cameras also make it possible to observe behaviors, to calculate event duration and to record the direction of all animals. This leads to the question of whether a wider use of permanent recording video monitoring would preferable to that of camera traps. However, this protocol is costly in terms of the time needed to analyze video data. Staring at a screen where nothing moves strains the eyes and diminishes the observer's attention to a point where he or she may miss an event. To reduce the time investment and improve the efficiency of this method, we tested whether the internal motion sensor (post‐recording sensor) of the video camera server could automatically detect events. Unfortunately, attaining a level of video sensitivity that would allow a systematic detection of events would entail recording on a practically permanent basis (*data not shown*). Moreover, video cameras are far more expensive than camera traps (based on the cost of devices used in our study, 59,056 € for video monitoring composed of 16 video cameras, 16 IR spots, two recorder servers, and cabling versus 15,000 € for 20 Reconyx HC600 including batteries, padlocks, and security enclosures), and power failure through storms or cable breakage could lead to the loss of several weeks of data. However, video camera systems are stolen less frequently than camera traps (Meek & Vernes, 2016). Neither technique is particularly effective for the differentiation between small mammals. For this reason, it is often necessary to physically trap these animals or use a white flash with camera traps (Meek & Pittet, 2014), but these methods are more intrusive (Glen et al., 2013). At equal cost, a video camera system would have a lower resolution than a camera trap and hence involve a greater difficulty in distinguishing species. However, video systems are surely less disturbing than camera traps, as the permanent recording emits continuous noises and lights, thus allowing habituation. Camera trap disturbances could also prevent animals from using wildlife crossings (Meek et al., 2014b).

Regardless of the advantages a video camera system may provide for animal detection, our financial and analytic investment leads us to recommend the use of camera traps in underpasses. However, we recommend the use of permanent recording video systems to periodically assess the efficiency of the detection methods by means of a constant and reliable control. Despite the high technological quality of current camera traps, we conclude that these systems may underestimate the numbers of animal events and therefore, the number of crossing events. This is especially relevant for small mammals in underpasses.

## CONFLICT OF INTEREST

None declared.

## AUTHOR CONTRIBUTIONS

None of the authors have any conflict of interest to declare, and all approve this version of the manuscript. They agree to be accountable for the aspects of the work that they conducted and will ensure that any questions related to the accuracy or integrity of any part of their work are appropriately investigated and resolved. JJ conceived the idea and design methodology; JJ collected the data; JJ, LP, and YH analyzed the data; JJ, LP, and YH led the writing of the manuscript. All authors actively contributed to the drafts and gave final approval for publication.
